# Beta-Propeller Protein-Associated Neurodegeneration (BPAN) Detected in a Child with Epileptic Spasms

**DOI:** 10.7759/cureus.5404

**Published:** 2019-08-16

**Authors:** Guneet Kaleka, M. Eileen McCormick, Anant Krishnan

**Affiliations:** 1 Internal Medicine, Olive View⁠ - University of California Los Angeles Medical Center, Sylmar, USA; 2 Pediatrics, Oakland University William Beaumont School of Medicine, Royal Oak, USA; 3 Radiology, Oakland University William Beaumont School of Medicine, Royal Oak, USA

**Keywords:** beta-propeller protein-associated neurodegeneration, bpan, wdr45, wipi4.

## Abstract

This report discusses a 13-year-old girl diagnosed with beta-propeller protein-associated neurodegeneration (BPAN). BPAN is an X-linked neurodegeneration disorder associated with a mutation in the WDR45 gene. It typically presents in childhood with encephalopathy, developmental delay, and seizures. Following an initial static phase, these symptoms then progress to dementia, dystonia, and parkinsonism in early adulthood. Our child initially presented with epileptic spasms, global developmental delay, speech delay, hypotonia, spasticity, scoliosis, and gait disturbance. While these symptoms remained unchanged in early childhood, they depicted accelerated deterioration at age 12-13 rather than in adulthood. Her diagnosis was made based on her clinical presentation and review of imaging that led to specific genetic testing confirming the condition. The imaging findings were of markedly low signal on gradient T2* sequences in the globus pallidus and substantia nigra and T1 hyperintensity in the substantia nigra, with associated diffuse brain volume loss. Unlike other cases reported in the literature, there was no classic area of central hypointensity on T1 imaging in the substantia nigra.

## Introduction

Neurodegeneration with brain iron accumulation (NBIA) encompasses a diverse group of inheritable neurodegenerative conditions that occur due to abnormal iron accumulation within the brain. The umbrella term of NBIA includes the diagnoses of pantothenate kinase-associated neurodegeneration (PKAN), phospholipase-associated neurodegeneration (PLAN), fatty acid hydroxylase-associated neurodegeneration (FAHN), mitochondrial protein-associated neurodegeneration (MPAN), Kufor Rakeb syndrome, aceruloplasminemia, neuroferritinopathy, and static encephalopathy of childhood with neurodegeneration in adulthood (SENDA), now renamed beta-propeller protein-associated neurodegeneration (BPAN) [1]. BPAN is an X-linked condition that occurs as a result of mutations in the gene WDR45 which encodes the WD repeat domain phosphoinositide-interacting protein 4 (WIPI4) [2,3]. Most affected patients are females as males are likely more severely affected and may not survive to birth.

As noted previously, BPAN classically presents in early childhood with encephalopathy, global developmental delay, and seizures, including petit mal, grand mal, atonic, or myoclonic seizures [2]. This is followed by a static period that then progresses to parkinsonism, dementia, and spasticity in early adulthood [2,4]. Treatment is focused on symptomatic relief, including medications for parkinsonism and seizure activity. Neuroimaging in BPAN patients reveals T2 and T2* hypointensity in the substantia nigra and globus pallidus with associated T1 hyperintensity in the substantia nigra that can extend to the globus pallidus [5]. A band of central low signal within this T1 hyperintensity has been described as pathognomonic for BPAN. We discuss a 13-year-old girl presenting with an initial static global developmental delay course and epileptic spasms who depicts a previously undescribed early progression in symptoms with the development of tonic seizures, and decline in gross and fine motor functions in early adolescence. This case was unique in the severe initial presentation, and the accelerated disease progression. 

## Case presentation

A 13-year-old girl who initially presented with epileptic spasms, sensorineural hearing loss, cognitive impairment, and global developmental delay was seen at our institution for epilepsy. She had extensive workup at multiple institutions without a unifying diagnosis for her symptoms. However, recently, after relatively static symptoms for four years, she demonstrated a deterioration in her symptoms, including a decline in fine and gross motor function and the development of brief tonic seizures. On examination, aphasia, oral motor apraxia, rotoscoliosis, spasticity, and brisk reflexes were noted. EEG documented frequent epileptic discharges with intermittent bursts of semi-rhythmic theta slowing in addition to clusters of, as well as, several isolated brief tonic seizures with diffuse onset.

**Figure 1:**
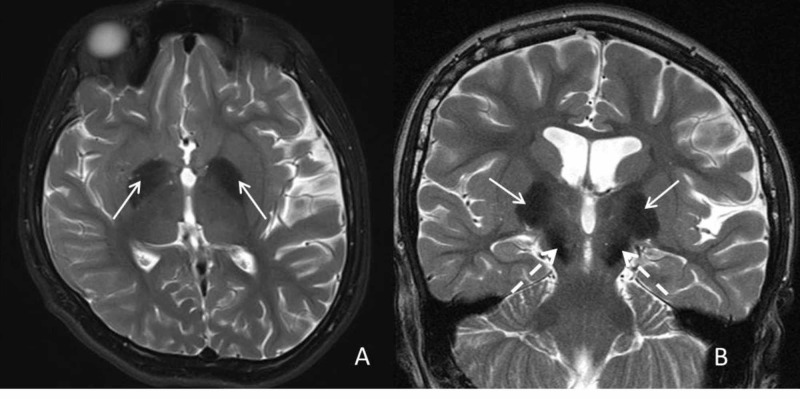
Axial (A) and Coronal (B) T2 weighted images Axial (A) and Coronal (B) T2 weighted images reveal low T2 signal at age 13 in the globus pallidus (arrows) and substantia nigra (interrupted arrows).

**Figure 2:**
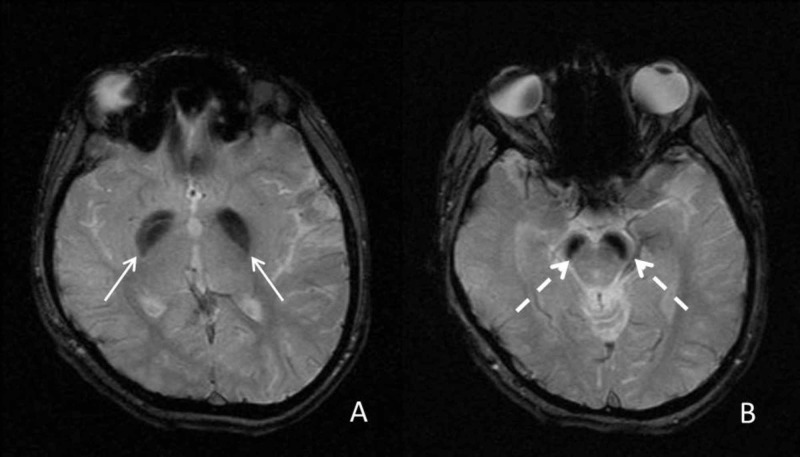
Axial gradient T2* images Axial gradient T2* images demonstrate the marked low signal in the globus pallidus (arrows) and substantia nigra (interrupted arrows).

**Figure 3:**
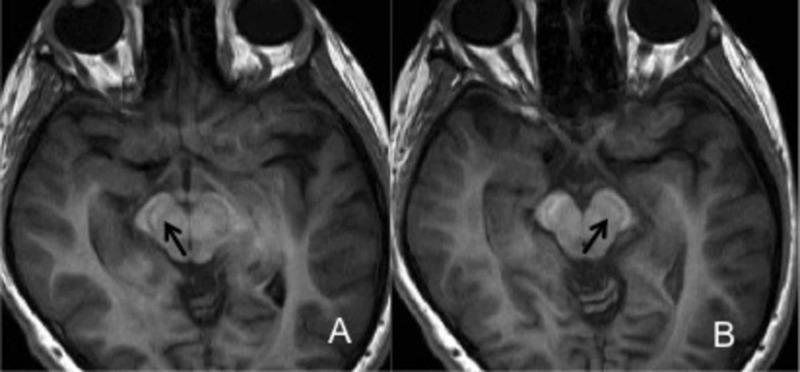
Axial 3D spoiled gradient T1 weighted images Axial 3D spoiled gradient T1 weighted images demonstrate mildly increased T1 signal within the substantia nigra (black arrows). Please note the head is a little tilted accounting for the side to side asymmetry.

She had undergone multiple imaging studies dating back to the age of two. MRIs performed at ages seven and eight demonstrated decreased T2 signal in the globus pallidi and substantia nigra. These changes were reportedly not seen at age two. MRI at our institution confirmed the low T2 signal (Figures 1A, 1B). In addition, on the dedicated gradient T2* imaging, significant hypointensity was seen in the globus pallidi and substantia nigra (Figures 2A, 2B). There was also supratentorial and infratentorial volume loss. Additionally, mildly increased T1 signal was also seen in the substantia nigra (Figures 3A, 3B) and globus pallidi. Based on the imaging findings, NBIA and mitochondrial disorders (given the cerebellar volume loss and history) were considered. The child underwent a targeted genetic panel, which revealed a heterozygous pathogenic variant in the WDR45 gene c.868C>T (p.Gln290Ter). Treatment is focused on symptomatic relief with clobazam, baclofen, levetiracetam, and a mitochondrial cocktail, including riboflavin, multivitamin, levocarnitine, coenzyme Q10, and vitamin C. The patient also continues to engage in physical and occupational therapy to improve functional ability and quality of life. 

## Discussion

BPAN is caused by mutations in the WDR45 gene, which encodes for the WIPI4 protein [2]. Members of the WIPI protein family are a part of many cellular processes, including cell cycle progression, apoptosis, gene regulation, and signal transduction. Mutations in the WDR45 gene impede autophagy, a process that helps prevent excess iron accumulation. Dysfunction leads to excess accumulation of iron in the brain, particularly in the basal ganglia [2]. The diagnosis of BPAN is made through the identification of a pathologic variant of the WDR45 gene using either gene-targeting sequencing or genomic testing [3]. As discussed previously, BPAN classically presents with global developmental delay, ataxia, and seizures in childhood. These symptoms subsequently undergo a static phase, ultimately progressing to parkinsonism and spasticity in adulthood. In this case, our patient depicted classical symptoms of intellectual impairment, gait abnormalities, and seizures attributed to BPAN at a young age. However, uniquely, she showed an accelerated deterioration in her symptoms with the development of tonic seizure activity, and a decline in motor function in early adolescence.

Characteristic neuroimaging findings in NBIA are a result of iron deposition, which, depending on the subtype, can occur at different locations though with a propensity for the basal ganglia [1]. As noted previously, in the case of BPAN, classically T2 and T2* hypointensity in the substantia nigra and globus pallidus are described with associated T1 hyperintensity in the substantia nigra that can extend to the globus pallidus [5]. While a band of central low signal within this T1 hyperintensity has been described as pathognomonic for BPAN, it was not distinctly seen in our patient [6]. In our patient, low T2 signal was seen by age seven but was best characterized at age 12, when she underwent additional T2* imaging. Gradient T2* sequences (or susceptibility-weighted imaging) are particularly helpful in detecting iron deposition. While we considered other causes for the low T2 signal such as Wilson’s disease and Fahr’s disease, the marked low T2* changes were felt more consistent with iron deposition. Among the NBIA disorders, PKAN was felt less likely given the lack of the ‘eye of the tiger’ abnormality and pigmentary retinopathy, though some patients with PKAN may not possess either of these findings. Similarly, while there was some volume loss, including in the cerebellum, it was not as dominant as typically seen in PLAN. There is some overlap with some of the other subtypes like MPAN (described to have increased T2 signal involving the medial medullary lamina) [6]. As a result, it may be difficult to definitively suggest the exact subtype of NBIA on imaging and confirmatory genetic testing is necessary for making the final diagnosis. 

Brain atrophy, in this patient, as well as, signal abnormalities were relatively stable from age seven to 13, which suggests that the changes occurring in the patient’s brain also underwent a static period as reflected in her stable clinical symptoms.

## Conclusions

BPAN is an X-linked type of NBIA that occurs due to de novo mutations in the WDR45 gene. Clinically, patients suffer from cognitive impairments early in life, followed by a static phase that progresses to sudden onset spasticity and parkinsonism later in life. Our child is unique in her accelerated clinical deterioration in early adolescence in juxtaposition to the previously described progression in adulthood. Neuroimaging characteristically depicts low T2 and marked low T2* signal in the globus pallidi and substantia nigra, the absence of the ‘eye of the tiger’ sign, as well as, T1 hyperintensity in the globus pallidus and substantia nigra with volume loss diffusely in the brain. The characteristic MRI and clinical appearance is strongly suggestive of this condition and can help direct the referring pediatric specialist to obtain the appropriate genetic testing. 
